# Adaptive deep brain stimulation in a freely moving parkinsonian patient

**DOI:** 10.1002/mds.26241

**Published:** 2015-05-21

**Authors:** Manuela Rosa, Mattia Arlotti, Gianluca Ardolino, Filippo Cogiamanian, Sara Marceglia, Alessio Di Fonzo, Francesca Cortese, Paolo M. Rampini, Alberto Priori

**Affiliations:** ^1^ Fondazione IRCCS Ca’ Granda Ospedale Maggiore PoliclinicoMilanItaly; ^2^ Università degli Studi di MilanoMilanItaly; ^3^ Università la Sapienza di RomaPolo PontinoLatinaItaly

The future of deep brain stimulation (DBS) for Parkinson's disease (PD) lies in new closed‐loop systems that continuously supply the implanted stimulator with new settings obtained by analyzing a feedback signal related to the patient's current clinical condition.^1^ The most suitable feedback for PD is subthalamic local field potential (LFP) activity recorded from the stimulating electrode itself.^2^, ^3^, ^4^ This closed‐loop technology known as adaptive DBS (aDBS) recently proved superior to conventional open‐loop DBS (cDBS) in patients with PD.^2^

No studies have yet tested aDBS in freely moving humans for a prolonged time. This information is an essential prerequisite for developing new implantable aDBS devices for chronic PD treatment.

In this single‐case study, we tested whether a portable DBS device we developed is suitable to compare the clinical benefit in a freely moving PD patient induced by either aDBS or cDBS. To do so, after a first experimental session for extracting patient settings to personalize the aDBS algorithm, we treated a blinded patient (51 y old, male, 8 y PD history) with cDBS and aDBS in two separate experimental sessions each lasting 120 min, 5 and 6 d, respectively, after DBS electrode implant. To ensure reliable results, the patient underwent repeated clinical assessments every 20 min (T1‐T5) by two independent blinded neurologists through Unified Parkinson's Disease Rating Scale (UPDRS) III subsections and Rush Dyskinesia Rating Scale (see Supplemental Data for details).

The aDBS portable device we used was equipped with an ad hoc algorithm that analyzed patient's LFP beta band power (13‐17 Hz) and adapted voltage stimulation linearly each second (Fig. 1A).

**Figure 1:**
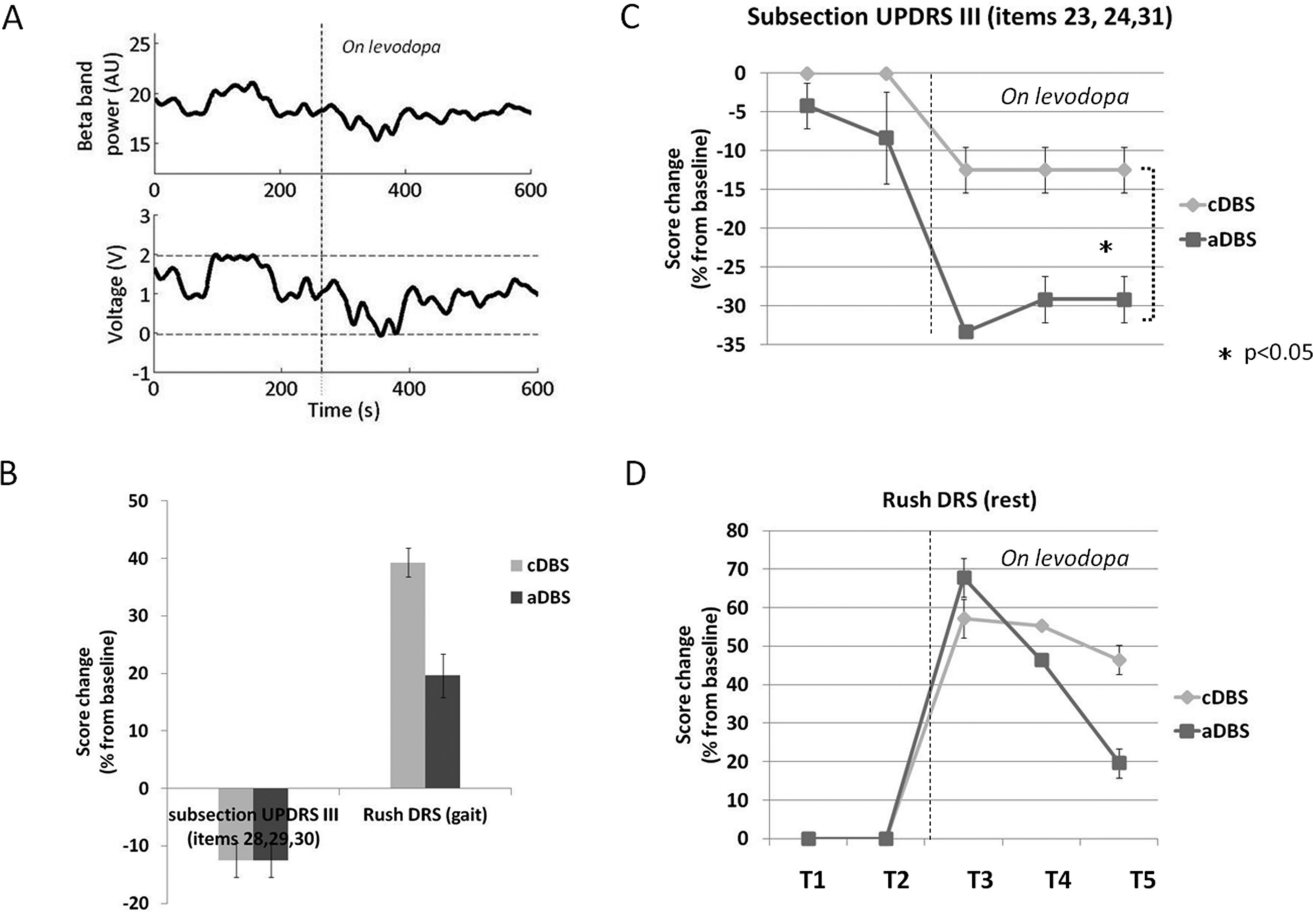
(**A**) Sample of aDBS functioning lasting 10 min. Upper panel, the local field potential (LFP) beta band (13‐17 Hz) power and below the stimulation voltage. The dotted line represents the time levodopa (l‐dopa) took to achieve its clinical effect. The voltage delivered by aDBS followed the beta‐band changes: When l‐dopa reduced beta‐band LFP activity, the voltage linearly diminished. (**B**) Clinical results for axial symptoms and dyskinesias during gait. Mean Unified Parkinson's Disease Rating Scale (UPDRS) III subsection (items 28, 29, 30) and mean Rush Dyskinesias Rating Scale (DRS) (during gait) percentage score changes from baseline evaluated at T5 (120 min after the experiment began) for cDBS and aDBS. Assessment at T5 showed that the patient's axial symptoms improved to a similar extent after aDBS and cDBS, but dyskinesias during gait reduced more during aDBS than during cDBS. (**C**) Clinical results for bradykinesia. Mean changes from baseline in the UPDRS III subsection (items 23, 24, 31) percentage score changes from baseline for the upper limb contralateral to the stimulation side for cDBS and aDBS from T1 to T5. The UPDRS III subscore improved significantly more during aDBS than during cDBS (Wilcoxon matched pairs test; *P < 0.05). (**D**) Clinical results for dyskinesias at rest. Mean Rush DRS (at rest) percentage score changes from baseline for cDBS and aDBS from T1 to T5. Except at T3, aDBS induced a lower mean Rush DRS increase than cDBS (Wilcoxon matched pairs test; *P* > 0.05) (see Supplemental Data for data analysis details).

The patient during aDBS experienced a more stable condition than during cDBS, with better control of symptoms and dyskinesias over time (Fig. 1; video 1). In particular, aDBS and cDBS improved patient's axial symptoms to a similar extent (Fig. 1B), but compared with cDBS, aDBS significantly improved his main symptom, bradykinesia (Fig. 1C). aDBS did not elicit side effects and was well tolerated.

Because we evaluated the patient a few days after surgery when he probably manifested a stunning effect,^5^ the aDBS‐ and cDBS‐induced improvements were lower than those reported by others in follow‐up DBS studies.^6^ A major clinical achievement was that compared with cDBS, aDBS greatly reduced the patient's dyskinesias during gait and at rest (Fig. 1B; Fig. 1D). Presumably it did so because we designed the adaptive algorithm to avoid dyskinesias related to hyperstimulation: when l‐dopa reduced beta‐band LFP activity, the voltage linearly diminished, avoiding hyperstimulation.

Our results, besides corroborating findings reported by Little and colleagues^2^ showing that aDBS promises to be more efficient and effective than cDBS, expand them for two important reasons. First, we tested aDBS for a longer observation time than Little et al., and in a more ecological condition (freely moving patient). Second, the personalized algorithm continuously adapts stimulation settings according to LFP beta changes, instead of providing an on–off strategy.

The aDBS device we used here can assess large patient series in real clinical settings, testing different LFP‐based adaptive strategies other than those controlled by the beta activity to find the frequency that is more suitable to reflect patient clinical state.^7^

In conclusion, the approach and device we used proved eligible for prolonged use in a freely moving parkinsonian patient and disclosed new opportunities to study aDBS during patients’ daily activities, providing new insights into how this novel DBS strategy should improve patients’ quality of life. Although we await future studies to confirm our findings and to test other aDBS LFP‐based algorithms, our observation is a step toward developing a new generation of implantable aDBS devices for chronic treatment of PD.

## Video legend

**Video:** The video shows a section of patient clinical assessments performed 120 min after the experiment began (T5) during standard DBS (cDBS) on the left and during adaptive DBS (aDBS) on the right. Standard DBS was delivered at 2 V, 130 Hz, 60 µs; aDBS was delivered at a stimulation voltage that automatically changes according to the online LFP beta recording analysis (voltage range, 0‐2 V), 130 Hz, 60 µs. The video shows the patient during the execution of items 29, 23, 24, and 31 of unified parkinson's disease rating scale (UPDRS) III scale.


Manuela Rosa, MS,*^1,2^ Mattia Arlotti, MS,^1,3^ Gianluca Ardolino, MD,^1^ Filippo Cogiamanian, MD,^1^ Sara Marceglia, MS, PhD,^1^ Alessio Di Fonzo, MD, PhD,^1^ Francesca Cortese, MD,^1,2,4^ Paolo M. Rampini, MD,^1^ and Alberto Priori, MD, PhD^1,2^
^1^Fondazione IRCCS Ca’ Granda Ospedale Maggiore Policlinico, Milan, Italy
*^2^Università degli Studi di Milano, Milan, Italy*
*^3^Department of Electrical, Electronic and Information Engineering ‘Guglielmo Marconi’, Università di Bologna, Cesena, Italy*
*^4^Università la Sapienza di Roma, Polo Pontino, Latina, Italy*

## Author Roles

1. Research Project: A. Conception, B. Organization, C. Execution; 2. Statistical Analysis: A. Design, B. Execution, C. Review and Critique; 3. Manuscript Preparation: A. Writing the First Draft, B. Review and Critique.

M.R.: 1C, 2B, 3A

M.A.: 1C, 2B

G.A.: 1C, 3B

F.C.: 1C, 3B

S.M.: 2A, 2C

A.D.F.: 1C

F.C.: 1C

P.M.R.: 1A, 1B

A.P.: 1A, 1B, 3B

## Financial Disclosures

Stock ownership in medically related field: *Newronika s.r.l*: Filippo Cogiamanian, Sara Marceglia, Paolo M Rampini, Alberto Priori

Consultancies: None

Advisory boards: None

Partnerships: None

Honoraria: None

Grants: *Italian Ministry of Health*: Sara Marceglia, Alberto Priori; *Fondazione IRCCS Ca’ Granda, Ospedale Maggiore Policlinico, Milan, Italy*: Alberto Priori

Intellectual Property Rights: *MI2010A001265*: Alberto Priori; *US11766401‐070621*: Alberto Priori; *EP1940508*: Alberto Priori; *US8078281*: Alberto Priori. *EP2328655*: Sara Marceglia; *EP2155323*: Filippo Cogiamanian; Sara Marceglia, Alberto Priori

Expert testimony: None

Employment: *Fondazione IRCCS Ca’ Granda, Ospedale Maggiore Policlinico, Milan, Italy*: Filippo Cogiamanian, Gianluca Ardolino, Alessio Di Fonzo, Paolo M Rampini; Università la Sapienza di Roma, Polo Pontino, Latina, Italy: Francesca Cortese. Universita' degli Studi di Milano, Italy: Alberto Priori

Contracts: *Fondazione IRCCS Ca’ Granda, Ospedale Maggiore Policlinico, Milan, Italy*: Sara Marceglia

Royalties: None

Others: None

## Supporting information

Additional supporting information may be found in the online version of this article at the publisher's web‐site.

**Video legend:** The video shows a section of patient clinical assessments performed 120 min after the experiment began (T5) during standard DBS (cDBS) on the left and during adaptive DBS (aDBS) on the right. Standard DBS was delivered at 2 V, 130 Hz, 60 µs; aDBS was delivered at a stimulation voltage that automatically changes according to the online LFP beta recording analysis (voltage range, 0‐2 V), 130 Hz, 60 µs. The video shows the patient during the execution of items 29, 23, 24, and 31 of UPDRS III scale.

Supporting Information Figure 1

Supporting Information
