# Supplementary figures and images for: Adaptive deep brain stimulation in a freely moving parkinsonian patient
Source: Mov Disord. 2015 May 21;30(7):1003–5. doi: 10.1002/mds.26241 (PMC5032989; doi:10.1002/mds.26241)

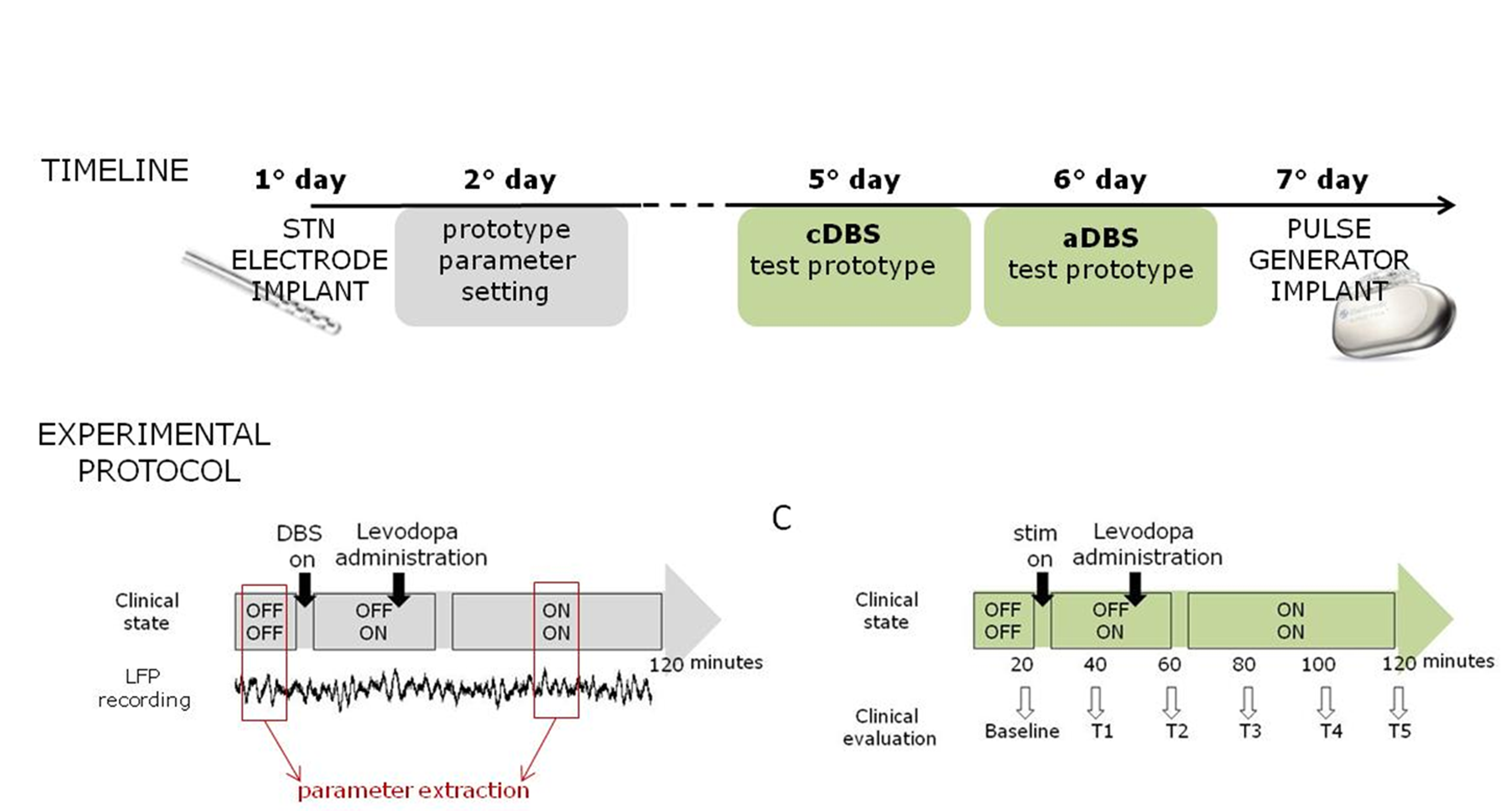

Supplement: Supplementary file 2 — Supporting Information Figure 1 [file MDS-30-1003-s002.tif]
