# Supplementary material for: Adaptive deep brain stimulation in a freely moving parkinsonian patient
Source: Mov Disord. 2015 May 21;30(7):1003–5. doi: 10.1002/mds.26241 (PMC5032989; doi:10.1002/mds.26241)
Supplement: Supplementary file 3 — Supporting Information [file MDS-30-1003-s001.docx]

**Supplementary materials**

*Case report*

The patient was a 51-year-old man who had suffered from PD for 8 years. Before DBS surgery the neurological examination in the on medication state showed moderate arm and leg dyskinesias, no resting tremor, rigidity or postural instability, mild-to-moderate bradykinesia, and slight hypophonia.

The patient was bilaterally implanted in the subthalamic nucleus (STN) with DBS electrodes (model 3389 Medtronic, Minneapolis, USA)^1^. Electrodes were externalized to allow LFP recordings before the electrodes were connected to the subcutaneous pulse generator (model 37601 Medtronic, Minneapolis, USA) implanted in a second operative procedure 7 days later (Fig.S1). The patient gave his informed consent to participate in the study which was approved by the institutional review board.

*Adaptive DBS device*

The aDBS prototype is a portable device (12 x 7 x 2.5 cm; 150 g) with a single input channel for LFP recording and a single output channel for stimulation (Arlotti M et al., An External Portable Adaptive Deep Brain Stimulation (aDBS) Device for Clinical Research, submitted). The system is programmable to include the individual patient’s LFP features. The device has two operating modes for stimulation, cDBS and aDBS, and one calibration mode for extracting the individual patient’s neurophysiological parameters. Since ample evidence suggests that LFP beta band in PD correlates with patient’s clinical state with and without treatment (either medication and stimulation)^2^ we developed an aDBS algorithm that used beta band as feedback control. The aDBS algorithm recognizes the patient’s specific LFP beta-band oscillations related to two clinical experimental states (levo off- stim off: patient without medication and stimulation; levo on – stim on: patient with medication and stimulation) and linearly adapts stimulation voltage each second according to beta band changes.

*Experimental protocol*

Two days after DBS electrode implant, after overnight withdrawal of antiparkinsonian medication, the aDBS device was used in the calibration mode (Fig. S1). As a first step, we defined the DBS voltage for cDBS. A neurologist indicated the right stimulation side and contact 1 from the clinical outcomes obtained during surgery. The voltage selected was the highest stimulation intensity that induced therapeutic benefits without adverse effects (2 V)^3^. The evolution of the voltage threshold for adverse effects carried out by the same neurologist according to DBS stimulation setting best practices.

Afterwards, the aDBS prototype in calibration mode recorded LFP from the contacts 0-2 , and extracted the beta band power in the levo-off stim-off and the levo-on stim-on clinical states. After a 20-minute local field potential (LFP) recording at rest (levo off – stim off), stimulation was turned on and LFPs were recorded for a further 20 minutes (levo off – stim on) at the voltage previously selected. Continuing LFP recording, we then gave the patient his usual levodopa dose and waited for the drug to achieve its clinical effect (about 30 min, levo on – stim on). With the patient under this condition, we then continuously recorded LFPs for a further 40 minutes (Fig. S1).

Five and six days after DBS electrode implant we tested the prototype in cDBS and aDBS modes for 120 minutes. The patient was unaware of the DBS type used. cDBS was delivered at 130 Hz, 60 µs and 2V. aDBS was delivered at 130 Hz, 60 µs and the personalized algorithm automatically adapted the voltage according to the online LFP recording analysis. In aDBS mode the stimulation voltage ranged from 0 and to 2V. The clinical assessment focused on bradykinesia, the patient’s main parkinsonian symptom, and dyskinesias: a blinded neurologist rated Unified Parkinson’s Disease Rating Scale (UPDRS) III subsection (items 23, 24, 31) score for the upper limb contralateral to the stimulation side, and the Rush Dyskinesias Rating Scale (DRS) at rest. The first clinical evaluation was performed in the stim off – levo off condition (baseline) and subsequently was repeated 5 times (T1-T5), every 20 minutes for all the 120 minute long experiment. At baseline and at the last clinical evaluation (T5), the neurologist evaluated the axial symptoms through the UPDRS III subsection (items 28, 29, 30) score and the Rush DRS during gait. These assessments were also video recorded and blindly rated by a neurologist not belonging to the research team.

*Data analysis*

After verifying that the scale scores rated by the two neurologists were consistent (Mann Withney U test for dependent samples, p<0.05), we averaged the two assessments by the two neurologists, and used them for further analysis. Clinical improvements were expressed as percentage changes from baseline normalized to the total scale score. To evaluate the clinical improvements during aDBS and cDBS, a Wilcoxon matched pairs test (p<0.05) was used to compare the score percentage changes for the 5 timepoints (T1- T5).

**Figure legend**

**Figure S1.** **Timeline and experimental protocol.** Above the experimental session timeline 2, 5 and 6 days after electrode implant; below (on the left) the experimental protocol undertaken 2 days after electrode implant for extracting patient settings to personalize the algorithm for the adaptive deep brain stimulation (aDBS) prototype. Below (on the right) the experimental protocol performed 5 and 6 days after electrode implant to test prototype functioning and the clinical effectiveness of conventional DBS (cDBS) and aDBS. Motor symptoms were assessed first in the stim off-levo off condition (baseline) and assessment was repeated 5 times (T1-T5), every 20 minutes throughout the 120-minute experiment.

**Refereces**

1. Rampini PM, Locatelli M, Alimehmeti R, et al. Multiple sequential image-fusion and direct MRI localisation of the subthalamic nucleus for deep brain stimulation. J Neurosurg Sci 2003;47(1):33-39.

2. Rosa M, Giannicola G, Marceglia S, Fumagalli M, Barbieri S, Priori A. Neurophysiology of deep brain stimulation. Int Rev Neurobiol 2012;107:23-55.

3. Little S, Pogosyan A, Neal S, et al. Adaptive deep brain stimulation in advanced Parkinson disease. Ann Neurol 2013;74(3):449-457.
